# Mitochondrial dysfunction in long COVID: mechanisms, consequences, and potential therapeutic approaches

**DOI:** 10.1007/s11357-024-01165-5

**Published:** 2024-04-26

**Authors:** Tihamer Molnar, Andrea Lehoczki, Monika Fekete, Reka Varnai, Laszlo Zavori, Szabina Erdo-Bonyar, Diana Simon, Tímea Berki, Peter Csecsei, Erzsebet Ezer

**Affiliations:** 1 Department of Anaesthesiology and Intensive Care, Medical School, University of Pecs, Pecs, Hungary; 2 Doctoral College, Health Sciences Program, Semmelweis University, Budapest, Hungary; 3 Department of Haematology and Stem Cell Transplantation, National Institute for Haematology and Infectious Diseases, South Pest Central Hospital, 1097 Budapest, Hungary; 4 Department of Public Health, Semmelweis University, Budapest, Hungary; 5 Department of Primary Health Care, Medical School University of Pecs, Pecs, Hungary; 6 Salisbury NHS Foundation Trust, Salisbury, UK; 7 Department of Immunology and Biotechnology, Medical School, University of Pecs, Pecs, Hungary; 8 Department of Neurosurgery, Medical School, University of Pecs, Ret U 2, 7624 Pecs, Hungary

**Keywords:** Long COVID, Mitochondrial dysfunction, Chronic fatigue, Post-infectious syndromes, Oxidative stress, Therapeutic strategies, Metabolic disturbances

## Abstract

The COVID-19 pandemic, caused by the SARS-CoV-2 virus, has introduced the medical community to the phenomenon of long COVID, a condition characterized by persistent symptoms following the resolution of the acute phase of infection. Among the myriad of symptoms reported by long COVID sufferers, chronic fatigue, cognitive disturbances, and exercise intolerance are predominant, suggesting systemic alterations beyond the initial viral pathology. Emerging evidence has pointed to mitochondrial dysfunction as a potential underpinning mechanism contributing to the persistence and diversity of long COVID symptoms. This review aims to synthesize current findings related to mitochondrial dysfunction in long COVID, exploring its implications for cellular energy deficits, oxidative stress, immune dysregulation, metabolic disturbances, and endothelial dysfunction. Through a comprehensive analysis of the literature, we highlight the significance of mitochondrial health in the pathophysiology of long COVID, drawing parallels with similar clinical syndromes linked to post-infectious states in other diseases where mitochondrial impairment has been implicated. We discuss potential therapeutic strategies targeting mitochondrial function, including pharmacological interventions, lifestyle modifications, exercise, and dietary approaches, and emphasize the need for further research and collaborative efforts to advance our understanding and management of long COVID. This review underscores the critical role of mitochondrial dysfunction in long COVID and calls for a multidisciplinary approach to address the gaps in our knowledge and treatment options for those affected by this condition.

## Introduction

The COVID-19 pandemic, triggered by the emergence of the novel coronavirus SARS-CoV-2, has precipitated an unprecedented global health crisis, the repercussions of which continue to resonate worldwide. While a significant proportion of those infected with COVID-19 recover, a startling observation has emerged: more than 70% of survivors experience lingering symptoms 4 months post-infection, giving rise to what is now recognized as “Long COVID Syndrome” or post-acute sequelae SARS-CoV-2 infection (PASC) [1–12]. This term encapsulates the enduring or emerging signs and symptoms that persist for weeks or months following the acute phase of COVID-19. Chronic fatigue emerges as the most frequently reported and debilitating symptom among survivors, as evidenced by numerous cross-sectional and cohort studies [13–17]. Individuals grappling with long COVID also report an extensive spectrum of symptoms in addition to chronic fatigue, including dyspnea, arthralgia, sleep disturbances, mood disorders such as depression and anxiety [18], headaches, dizziness, cognitive impairments colloquially termed as “brain fog,” and cardiac symptoms. The profound and diverse nature of these symptoms significantly undermines daily functioning and diminishes quality of life, imposing considerable personal, societal, and economic challenges. The persistent symptomatology of long COVID indicates that the impact of COVID-19 extends well beyond the respiratory system, implicating multiple organ systems and bodily functions, and highlights an urgent need for a comprehensive understanding of its long-term consequences on human health.

Mitochondria, the cellular organelles known as the powerhouses of the cell, are central to this discussion. They play a crucial role in cellular energy production through the process of oxidative phosphorylation, in addition to their involvement in oxidative stress, apoptosis (programmed cell death), induction of cellular senescence, and the modulation of immune responses. The function of mitochondria is essential for maintaining cellular and systemic homeostasis. Disruptions in mitochondrial function can lead to a decrease in energy production, increased production of reactive oxygen species (ROS), and initiation of inflammatory pathways, all of which can contribute to the pathophysiology of various diseases. The significance of exploring mitochondrial dysfunction in long COVID lies in the potential overlap between the symptoms of this condition and known consequences of mitochondrial impairment [19, 20]. Understanding how SARS-CoV-2 infection may lead to mitochondrial dysfunction could provide valuable insights into the mechanisms underlying long COVID symptoms. This knowledge could pave the way for novel therapeutic approaches aimed at mitigating these symptoms by targeting mitochondrial health and function. Thus, examining the role of mitochondrial dysfunction in long COVID not only offers a promising avenue for research but also holds the potential to improve the management and treatment of individuals suffering from this debilitating condition.

## Evidence of mitochondrial dysfunction in long COVID

**Fig. 1:**
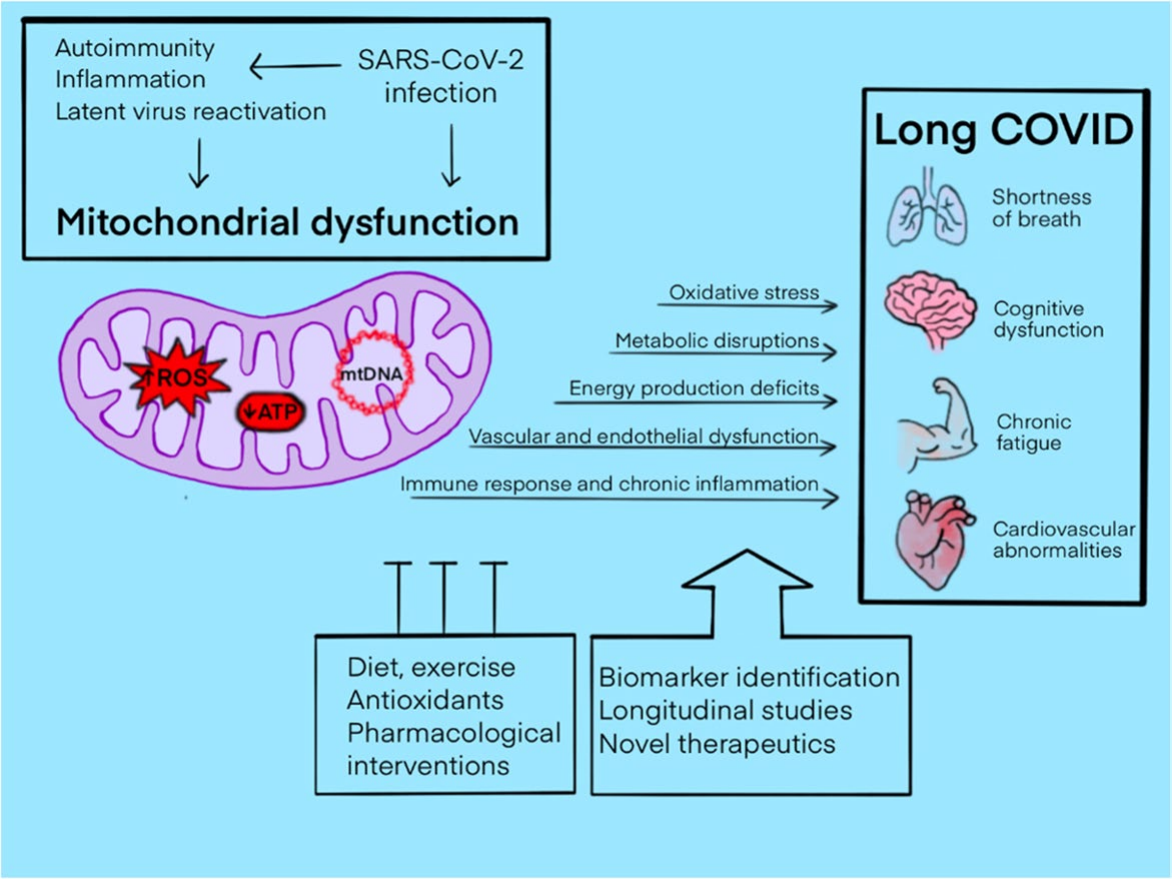
Interplay of mitochondrial dysfunction and long COVID: pathophysiological mechanisms and therapeutic approaches. This figure presents a comprehensive overview of mitochondrial dysfunction in long COVID, illustrating the relationship between mitochondrial impairment and the diverse symptoms of long COVID, as well as potential therapeutic interventions. COVID-19-related mitochondrial dysfunction leads to impaired cellular energy production, increased oxidative stress, and the induction of inflammatory responses. Mitochondrial dysfunction is causally linked to key symptoms associated with long COVID—cognitive disturbances (brain), fatigue and muscle weakness (muscle), breathlessness (lung), and cardiac symptoms (heart)—linked to mitochondrial dysfunction through mechanisms such as energy production deficits, oxidative stress, immune response dysregulation, metabolic disruptions, and vascular and endothelial dysfunction. Therapeutic strategies targeting mitochondrial health, including antioxidants, exercise, dietary modifications, and pharmacological interventions, may mitigate the aforementioned symptoms emphasizing the multifaceted approach required to address long COVID. The importance of further research into mitochondrial function as a potential therapeutic target for long COVID is emphasized, highlighting areas for future investigation such as biomarker identification, longitudinal studies, and the development of novel therapeutics

The emergence of long COVID as a distinct post-acute phase of SARS-CoV-2 infection has prompted investigations into its underlying pathophysiological mechanisms. Among the various avenues explored, evidence pointing towards mitochondrial dysfunction has garnered significant attention [19–23]. A growing body of research suggests that mitochondrial impairment may play a crucial role in the constellation of symptoms observed in long COVID patients [19–32] (Fig. 1). Several studies have documented mitochondrial dysfunction in individuals suffering from long COVID, providing insights into how this condition may perpetuate the diverse and persistent symptoms associated with the syndrome. For instance, research has identified abnormalities in mitochondrial respiration and bioenergetics and mitochondria-related gene expression in peripheral blood mononuclear cells (PBMCs) from long COVID patients [33–37]. These abnormalities are indicative of compromised mitochondrial energy production, which could underlie symptoms of fatigue and muscle weakness. Moreover, studies employing magnetic resonance spectroscopy (MRS) have observed alterations in muscle tissue and the brain indicative of mitochondrial dysfunction in long COVID sufferers [38–41], although data obtained in the heart are inconclusive [33, 42]. Such findings align with clinical reports of exercise intolerance and post-exertional malaise among these individuals [43].

### Biomarkers of mitochondrial dysfunction observed in long COVID

Further evidence of mitochondrial involvement in long COVID comes from biomarker studies. Elevated levels of circulating biomarkers associated with oxidative stress and mitochondrial damage, such as F2-isoprostanes, malondialdehyde, and reduced levels of antioxidants like coenzyme Q10, have been reported [20, 21, 44–48]. These biomarkers point towards oxidative stress as a contributing factor to mitochondrial dysfunction in long COVID. Additionally, genomic studies have identified expression changes in genes associated with mitochondrial function and the cellular response to viral infections in COVID-19 patients [49–52]. These changes may reflect direct viral effects on mitochondrial integrity and function or secondary effects related to the immune response to the infection. Collectively, these studies offer compelling evidence that mitochondrial dysfunction is a significant contributor to the pathophysiology of long COVID. By impairing energy production and exacerbating oxidative stress, mitochondrial dysfunction could explain the wide array of symptoms experienced by patients, from fatigue and muscle weakness to cognitive disturbances and beyond. This growing evidence base underscores the importance of further research into mitochondrial health as a potential target for therapeutic interventions in long COVID.

### Comparison with mitochondrial dysfunction in other post-infectious syndromes

The concept of post-infectious syndromes, where symptoms persist long after the acute phase of an infection has resolved, is not unique to COVID-19. Several other infections have been associated with chronic sequelae, where mitochondrial dysfunction has been proposed as a contributing factor. Understanding these conditions can provide valuable insights into the mechanisms that might also underlie long COVID. Here are a few notable examples.

#### Myalgic encephalomyelitis/chronic fatigue syndrome (ME/CFS)

ME/CFS is perhaps the most well-studied condition in the context of post-infectious syndromes and mitochondrial dysfunction. The symptoms of long COVID syndrome are very similar to those of ME/CFS [35, 53–56]. It is a chronic, complex disorder characterized by profound fatigue, sleep abnormalities, pain, and other symptoms that are worsened by exertion. The condition often follows viral infections, suggesting a post-infectious origin for some patients. Several studies have implicated mitochondrial dysfunction in ME/CFS, including evidence of structural changes and dysfunctional energy producing pathways, impaired ATP production, abnormalities in mitochondrial respiratory chain function, and elevated lactate levels suggestive of metabolic stress [57–67]. These findings parallel some of the mitochondrial abnormalities observed in long COVID patients, suggesting a common pathway of disease development. Mitochondria are a major source of reactive oxygen species (ROS) such as H_2_O_2_ and superoxide in the cell, making them extremely vulnerable to oxidative stress. In individuals with ME/CFS, elevated levels of peroxide and superoxide correlate with symptom severity [68, 69]. There is evidence that redox imbalance contributes to the pathogenesis of both (ME/CFS) and long COVID syndrome [70]. Proposed mechanisms underlying mitochondrial dysfunction associated with ME/CSF may include chronic immune activation and autoimmune responses that target mitochondrial components.

#### Post-treatment Lyme disease syndrome (PTLDS)

PTLDS refers to a condition where symptoms such as fatigue, pain, and cognitive impairment persist after treatment for Lyme disease, caused by the bacterium *Borrelia burgdorferi *[71]. While the exact mechanisms underlying PTLDS are not fully understood, it is possible that mitochondrial dysfunction may play a role. Research has shown alterations in mitochondrial morphology and function in patients with Lyme disease [72], which may contribute to the chronic symptoms experienced by some individuals after treatment. Likely mechanisms underlying mitochondrial dysfunction associated with PTLDS may involve lingering bacterial debris or immune complexes that continue to stimulate an immune response, affecting mitochondrial function.

#### Epstein-Barr virus (EBV) and other herpesviruses

Infections with EBV and other members of the herpesvirus family have been linked to chronic symptoms in some individuals, including fatigue and cognitive dysfunction [73–75]. These viruses encode proteins that can interfere with mitochondrial function, including inducing mitochondrial fragmentation and impairing mitochondrial bioenergetics [73, 76–78]. The chronic activation of the immune system in response to latent viral infection could also lead to sustained mitochondrial damage and dysfunction, contributing to the persistence of symptoms.

#### Chronic Q fever fatigue syndrome (QFS)

Chronic Q fever, caused by the bacterium *Coxiella burnetii*, can lead to Q fever fatigue syndrome (QFS), where patients experience severe fatigue, muscle pain, and concentration problems for years after the acute infection [79]. While specific studies on mitochondrial function in QFS are limited [80], the symptomatology suggests a potential role for mitochondrial dysfunction, similar to ME/CFS and other chronic conditions. Likely mechanisms include chronic inflammation triggered by *Coxiella burnetii*. Persistent immune activation may continue to disrupt mitochondrial function.

These examples illustrate that the association between chronic post-infectious symptoms and mitochondrial dysfunction is not unique to long COVID. The investigation into mitochondrial health in these conditions suggests a potential common pathway where viral or bacterial infections lead to mitochondrial damage, resulting in energy production deficits and oxidative stress, which in turn contribute to chronic symptoms. Understanding the mechanisms of mitochondrial impairment in these syndromes may offer insights into potential therapeutic targets and interventions for long COVID and other post-infectious conditions.

### Potential mechanisms of persistent mitochondrial dysfunction post-COVID-19

#### Long-lasting structural damage to mitochondria

During the acute phase of COVID-19, SARS-CoV-2 can directly interact with mitochondria, exploiting mitochondrial dynamics for virus proliferation [81, 82] and causing structural damage [32, 83–86]. This damage includes alterations in mitochondrial morphology, such as swelling and changes in size and number, driven by the effects of the virus on mitochondrial physiology. In theory, structural damage could impair mitochondrial function long-term, even after the virus has been cleared from the circulation [22, 85].

#### Persistent dysregulation of mitochondrial bioenergetics

Interaction between SARS-CoV-2 and mitochondrial components might lead to prolonged dysregulation of mitochondrial bioenergetics [29]. For example, viral proteins may interfere with the mitochondrial antiviral-signaling protein (MAVS), disrupting its normal function in the antiviral response and leading to chronic mitochondrial dysfunction [87, 88]. This persistent dysregulation could result from epigenetic modifications induced by the virus or from prolonged imbalances in proteins that regulate mitochondrial biogenesis and function.

#### Autoimmunity triggered by COVID-19

There is growing evidence that COVID-19 can trigger autoimmune responses [89–97] that may exacerbate or prolong mitochondrial dysfunction [98]. The immune system’s response to SARS-CoV-2 might include the development of autoantibodies that mistakenly target mitochondrial proteins, a phenomenon possibly driven by molecular mimicry [99]. These autoimmune responses can lead to continuous immune-mediated damage to mitochondria, maintaining a state of inflammation and mitochondrial impairment that persists well beyond the acute phase of the infection.

## Mechanisms linking mitochondrial dysfunction to long COVID symptoms

In the complex landscape of long COVID, mitochondrial dysfunction emerges as a prominent feature, potentially contributing to the diverse and persistent symptoms observed in patients.

One of the hallmark symptoms of long COVID is chronic fatigue, which can be directly linked to deficits in mitochondrial ATP production [100]. When mitochondrial function is compromised, ATP production is reduced, leading to energy deficits that manifest as profound and persistent fatigue [101, 102]. This energy shortfall impacts muscle function, cognitive processes, and overall physical capacity, significantly affecting quality of life. Impaired mitochondrial function and disruption of the electron transport chain promote the genesis of reactive oxygen species (ROS) leading to oxidative stress. Increased levels of ROS can cause damage to cellular structures, including lipids, proteins, and DNA, contributing to the tissue damage observed in long COVID [20, 31, 45, 46]. Oxidative stress is also implicated in aging and various chronic diseases [103], suggesting a common pathway through which mitochondrial dysfunction can exacerbate long COVID symptoms. Fatigue in long COVID bears resemblance to the fatigue experienced by heart failure patients, a condition where mitochondrial dysfunction has also been implicated [104, 105]. In heart failure, compromised mitochondrial efficiency significantly impairs cardiac function, directly contributing to symptoms of fatigue and limited exercise capacity [106–111]. Given the heart’s reliance on mitochondrial oxidative phosphorylation for its substantial energy needs, any reduction in this process can result in marked energy deficits. This mitochondrial inefficiency in heart failure parallels the potential role of mitochondrial dysfunction in exacerbating the fatigue observed in long COVID patients. Such similarities underscore a possible common pathway of disease impact through mitochondrial compromise, enhancing our understanding of long COVID’s debilitating effects.

Mitochondria play a crucial role in modulating immune responses and inflammation. Mitochondrial dysfunction and increased levels of mitochondria-derived ROS can activate Nf-KB and the NLRP3 inflammasome, leading to the production of pro-inflammatory cytokines and chronic inflammation. This inflammatory response can perpetuate tissue damage and contribute to the systemic symptoms of long COVID, such as fatigue and malaise [46, 48, 112]. The chronic activation of the immune system, driven by ongoing mitochondrial dysfunction, may also hinder recovery and prolong the duration of symptoms. Mitochondrial dysfunction affects various metabolic pathways, including glucose metabolism, lipid oxidation, and amino acid turnover. These disruptions can lead to metabolic imbalances that contribute to the symptoms of long COVID, such as weight changes, exercise intolerance, and nutritional deficiencies. The impact on metabolism further complicates the clinical picture of long COVID, making management and treatment more challenging.

In the context of COVID-19, accumulating evidence suggests that SARS-CoV-2 directly targets endothelial cells, highlighting a critical aspect of the virus’s pathogenicity [113–126]. Emerging evidence suggests that mitochondrial dysfunction may contribute to vascular issues seen in long COVID, including endothelial dysfunction and impaired blood flow. Mitochondria are involved in the regulation of endothelial cell function and vascular tone [127–137]. Mitochondrial dysfunction can lead to increased oxidative stress and inflammation within the vascular system, contributing to the development of endothelial dysfunction. This can result in a range of symptoms, including blood pressure variations, dizziness, and an increased risk of thrombosis, all of which have been reported in long COVID patients.

In expanding our understanding of the cellular targets SARS-CoV-2, it is crucial to consider not only the endothelium but also intestinal enterocytes, which express the ACE2 receptor utilized by the virus for cell entry [138, 139]. The infection of intestinal enterocytes can lead to significant clinical implications, including malabsorption, which may result in deficiencies in essential nutrients critical for mitochondrial function and overall energy metabolism. Moreover, persistent enterocyte dysfunction can perpetuate chronic inflammation, both locally within the gut and systemically [138, 139]. This ongoing inflammatory response may also exacerbate mitochondrial dysfunction, contributing to the energy deficits and general malaise.

In summary, mitochondrial dysfunction is intricately linked to the diverse symptoms of long COVID through its impact on energy production, oxidative stress, immune response, metabolic processes, and vascular health. Understanding these mechanisms is crucial for developing targeted interventions to mitigate the effects of long COVID and improve patient outcomes.

## Serum peroxiredoxin-3: a novel biomarker of mitochondrial dysfunction in long COVID

Peroxiredoxin-3 is a member of the peroxiredoxin family of antioxidant enzymes, which are crucial for reducing oxidative stress within cells. Specifically, PRDX3 is located in the mitochondria, where it plays a key role in detoxifying hydrogen peroxide (H_2_O_2_) and protecting cells from oxidative damage [140]. Recent research indicates that PRDX3 is responsible for removal of up to 90% of the H_2_O_2_ generated in mitochondria [141]. Given its mitochondrial localization and its role in defending against oxidative stress, PRDX3 is used as a marker for mitochondrial oxidative stress and dysfunction [142, 143].

The utility of PRDX3 as a mitochondrial biomarker lies in its ability to reflect changes in mitochondrial health and oxidative stress levels. Increased levels of PRDX3 in serum or other bodily fluids can indicate heightened oxidative stress within mitochondria, potentially signaling mitochondrial dysfunction. This makes it a valuable tool for studying oxidative stress and mitochondrial impairment in long COVID.

### Study objectives and methods

In light of the emerging significance of PRDX3, we aimed to investigate a possible association between serum PRDX3 levels measured in post-COVID patients and the characteristics of their fatigue syndrome. This objective was pursued to further understand the role of mitochondrial dysfunction in the persistence of fatigue symptoms in long COVID, providing insights into potential diagnostic and therapeutic strategies. We conducted a prospective, single-center cohort study at the University of Pécs Clinical Center, Hungary, collaborating closely with General Practitioners (GPs) within the university’s operational area. Patients who presented with persistent post-COVID symptoms at their GP’s office were referred to our outpatient clinic for a baseline visit. Inclusion criteria were as follows: (i) a minimum of 30 days since symptom onset at the time of the outpatient visit; (ii) confirmation of SARS-CoV-2 infection via a positive PCR or antigen test; (iii) presence of symptoms at the time of the outpatient appointment; and (iv) age 18 years or older. We excluded individuals with the following: (i) pre-existing malignant or active autoimmune diseases; (ii) ongoing immunosuppressive therapy; (iii) acute coronary syndrome; (iv) vaccination against SARS-CoV-2; and (v) any condition potentially confounding the assessment of fatigue or cognitive state. Based on the duration from acute SARS-CoV-2 infection onset to study enrollment (4–12 weeks vs. > 12 weeks), patients were stratified in accordance with the National Institute of Care and Health Excellence (NICE) guidelines [144]. Approved by the Hungarian Medical Research Council (IV/2505–3/2021/EKU), our study adhered to the ethical guidelines of the 1975 Declaration of Helsinki. All participants provided written informed consent.

Following our previous methodologies, fatigue was assessed using the Chalder Fatigue Scale (CFQ-11) [17, 145]. Patients reported their general condition over the past 3 days, with scores ranging from 0 to 3 via the Likert scoring system, enabling calculation of a global score (max 33) and subscales for physical (0–21) and psychological fatigue (0–12) [146, 147]. Severe fatigue was defined using a bimodal scoring system, with a score ≥ 4 indicating significant fatigue [146, 147]. The Post-COVID-19 Functional Status (PCFS) Scale measured the general impact of post-COVID symptoms on daily life, considering only symptoms present within the week prior to the baseline visit [146, 147]. Peripheral blood samples were collected, centrifuged, and the supernatant stored at − 80 °C until analysis. We measured antibodies against SARS-CoV-2 and PRDX3 concentrations using an ELISA kit from ABNOVA Corp., Taipei, Taiwan. Data were evaluated using SPSS (version 11.5; IBM, Armonk, NY, USA). The Kolmogorov–Smirnov test was applied to check for normality. To analyze demographic and clinical factors, the chi-square test was used for categorical data while the Student *t* test was applied to quantitative values. Non-normally distributed data were presented as median and interquartile range (25–75th percentiles) and were compared with the use of Mann–Whitney test. Correlation analysis was performed by calculating Spearman’s correlation coefficient (rho). After dichotomisation of the subjects based on age, binary logistic regression analysis was performed to explore predictors of various symptoms. The best cut-off value of predictors was determined based on ROC analysis. A *p*-value < 0.05 was considered statistically significant.

### Link between PRDX3 levels and long COVID symptoms

**Fig. 2:**
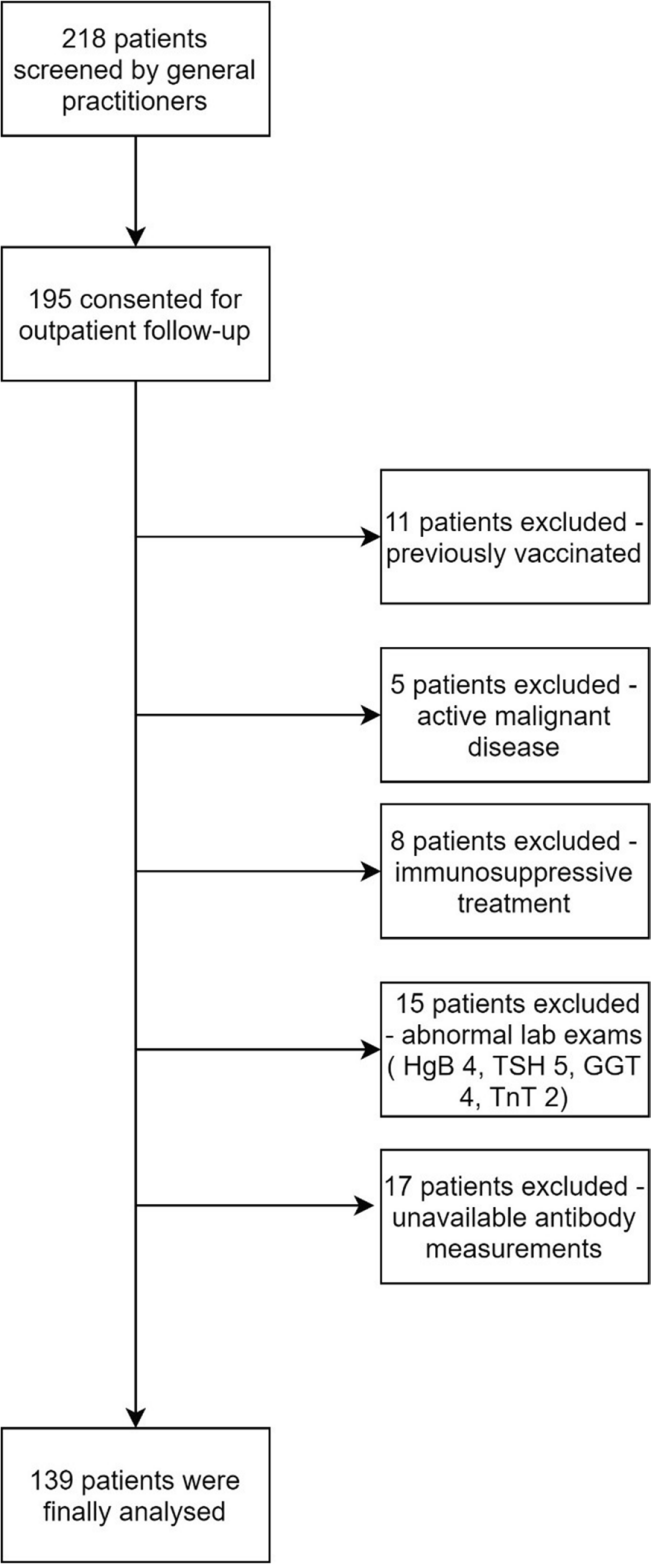
Patient selection chart

In our investigation into the potential role of levels as a biomarker for long COVID symptoms, we initially screened 218 patients, ultimately including 139 in the study after applying our exclusion criteria (Fig. 2). Table 1 shows the demographic and clinical characteristics as well as the systemic levels of anti-Spike (anti-S-Ig) and anti-Nucleocapsid (anti-NC-Ig) immunoglobulins of cohort according to the severity of post-COVID fatigue. Our analysis revealed a distinct pattern among those suffering from severe versus non-severe post-COVID fatigue. Notably, the total number of post-COVID symptoms (severe: 7, IQR: 4–10 vs. non-severe 2, 0–4, *p* < 0.001) as well as the number of symptoms related to acute SARS-CoV-2 infection reported by patients (severe: 7, IQR: 4–8 vs. non-severe: 5, 3–6, *p* = 0.001) were significantly higher in patients with severe fatigue when compared to those with less severe fatigue. Both anti-S-Ig (severe: 122 U/mL, IQR: 37–232 vs. non-severe: 189 U/mL, 99–474, *p* = 0.009) and anti-NC-Ig (severe: 43.4 U/mL, IQR: 17–101 vs. non-severe: 99 U/mL, IQR: 50–117, *p* = 0.002) antibody levels were significantly lower in the group of patients with severe fatigue than in those with non-severe fatigue.

**Table 1:** Demographic, clinical characteristics, and laboratory values of cohort according to the severity of post-COVID fatigue

	Total (*N* = 139)	Severe fatigue (*N* = 94)	Non-severe fatigue (*N* = 45)	*p-*value
Age, (ys)	50 (42–58)	48 (42–56)	55 (46–59)	0.057
Female, *N* (%)	90 (64.7%)	70 (74.5%)	20 44.4%)	0.001
BMI	26 (23–30)	26 (23–30)	27 (24–30)	0.542
Hospitalized, *N* (%)	50 (36%)	33 (35.1%)	17 (37.8%)	0.759
Medication, *N* (%)	51 (36.7%)	34 (36.2%)	17 (37.8%)	0.854
Favipiravir, *N* (%)	39 (28.1%)	24 (25.5%)	15 (33.3%)	0.338
Remdesevir, *N* (%)	12 (8.6%)	10 (10.6%)	2 (4.4%)	0.224
Steroid, *N* (%)	41 (29.5%)	26 (27.7%)	15 (33.3%)	0.492
PCFS	1 (1–2)	2 (1–2)	1 (0–1)	< 0.001
Chalder Fatigue Scale, Total	16 (12–20)	19 (16–21)	11 (9–13)	< 0.001
Chalder Fatigue Scale, Physical	13 (9–15)	14 (12–16)	7 (7–9)	< 0.001
Chalder Fatigue Scale, Psychological	4 (2–6)	4 (4–6)	4 (1–4)	< 0.001
Number of symptoms, index disease	6 (4–7)	7 (4–8)	5 (3–6)	0.001
CTss	9 (4–12)	9 (4–12)	9 (5–11)	0.547
O_2_-supplementation, *N* (%)	30 (21.6%)	21 (22.3%)	9 (20%)	0.754
Onset to follow-up time, days	65 (41–98)	60 (40–98)	71 (52–94)	0.292
Baseline WBC, G/L	6.3 (5–8)	6.4 (5–9)	6.3 (5–7)	0.758
Baseline creatinine, mmol/L	68 (57–78)	67 (57–76)	72 (58–79)	0.148
Baseline platelet, G/L	246 (212–297)	246 (218–299)	246 (208–281)	0.372
Baseline CRP, mg/L	1.5 (0.7–3.3)	1.7 (0.7–4)	1.3 (0.9–2)	0.187
Baseline D-dimer, µg/L	322 (220–491)	346 (221–510)	310 (201–436)	0.496
Baseline hsTroponin-T ng/L	4 (3–6)	3.7 (3–6)	4.5 (4–9)	0.016
Baseline AST, U/L	21 (17–25)	20 (16–25)	22 (18–29)	0.068
Baseline ALT, U/L	24 (16–38)	24 (16–38)	24 (16–36)	0.960
Baseline GGT, U/L	23 (15–47)	25 (15–47)	22 (15–40)	0.948
Number of post COVID symptoms	5 (3–8)	7 (4–10)	2 (0–4)	< 0.001
VAS score, Total	16 (10–20)	18 (14–21)	6 (0–15)	< 0.001
Anti-S Ig, U/mL	135 (43–323)	122 (37–232)	189 (99–474)	0.009
Anti-NC Ig, U/mL	58 (21–108)	43.4 (17–101)	99 (50–117)	0.002
Peroxiredoxin-3, ng/L	51 (41–62)	49.6 (40–63)	52 (43–61)	0.905

The categorical variables are presented as frequency (%), and the continuous variables are presented as median with interquartile range (IQR). The inter-group differences were assessed using chi-square test, Fisher exact test, and Mann–Whitney *U* test as appropriate in order to compare differences between non-severe and severe fatigue cases as per the CFQ-11 “caseness” definition for severe fatigue. *N* number, *BMI* body mass index, *PCFS* Post-COVID Functional Status Scale, *CTss* computer tomography severity score, *WBC* white blood cell count, *CRP* C-reactive protein, *AST* aspartate aminotransferase, *ALT* alanine aminotransferase, *GGT* gamma-glutamyl transferase, *VAS* visual analogue scale, *CFQ-11* Chalder Fatigue Scale, *COVID* coronavirus disease, *S-Ig* spike immunoglobulin, *NC-Ig* nucleocapsid immunoglobulin

**Fig. 3:**
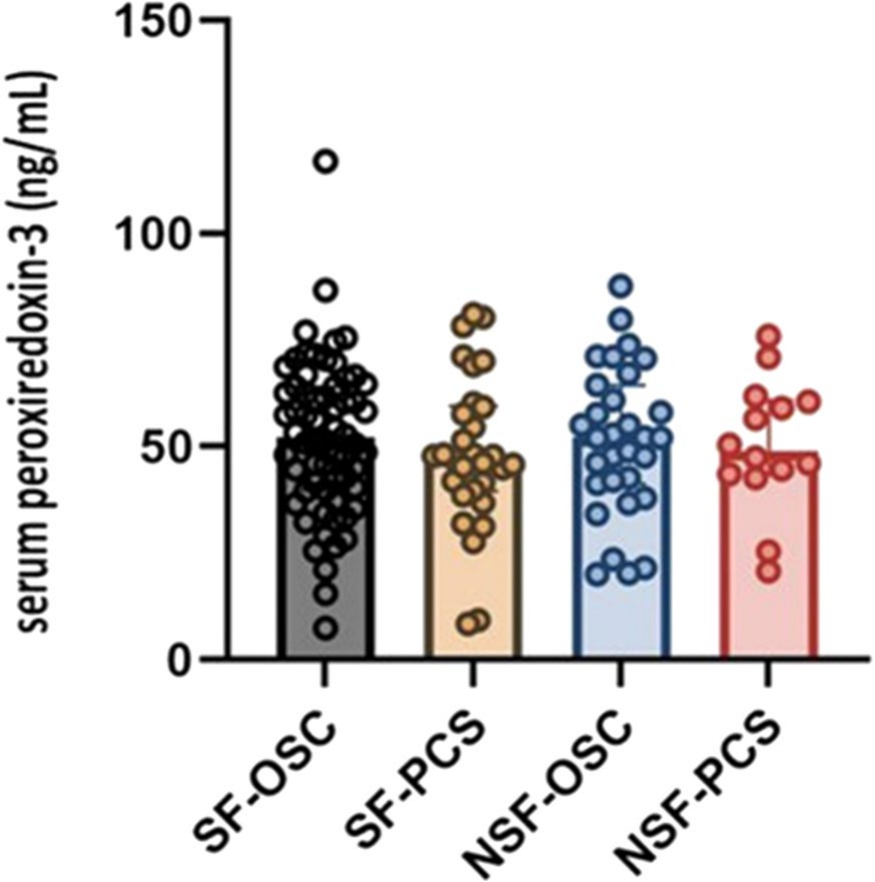
Association of serum peroxiredoxin-3 levels between patients with different degrees of fatigue observed in different phases of long COVID syndrome. SF-OSC, severe fatigue in patients with ongoing symptomatic COVID (*N* = 64); SF-PCS, severe fatigue in patients with post-COVID syndrome (*N* = 30); NSF-OSC, non-severe fatigue in patients with ongoing symptomatic COVID disease (*N* = 31); NSF-PCS, non-severe fatigue in patients with post-COVID syndrome (*N* = 14); OSC, patients who still have symptoms between 4 and 12 weeks after the start of acute symptoms; PCS, people who still have symptoms for more than 12 weeks after the start of acute symptoms. A total binary Chalder Fatigue Scale of 3 or less represents scores of those who have non-severe fatigue (NSF), with scores of 4 or more equating to severe fatigue (SF). Data are presented as individual dots and median with interquartile range. *Significant value (*p* < 0.05)

Intriguingly, when we examined the baseline serum PRDX3 levels in relation to the severity of post-COVID fatigue, we found no significant association. This absence of correlation extended to other parameters commonly associated with long COVID syndrome, such as scores on the Post-COVID-19 Functional Status (PCFS) Scale and the total number of reported symptoms (Fig. 3). These findings initially suggested that PRDX3 levels might not serve as a straightforward biomarker for the overall severity of long COVID symptoms. However, a deeper dive into the data uncovered a significant relationship between PRDX3 levels and the presence of dizziness among the study participants. Patients experiencing post-COVID dizziness had markedly higher PRDX3 levels compared to those without this symptom (median: 58.4 ng/ml; IQR: 48.0–66.9 vs 47.9; 39.8–60.4; *p* = 0.026). Individuals with post-COVID dizziness presented with more severe fatigue based on either PCFS, or each Chalder Fatigue (physical and psychological) scales (all *p* < 0.001). After dichotomization of patients under 50 years (*n* = 71), PRDX3 was also proved to be an independent predictor of dizziness (*n* = 15) after including other confounders such as age, gender, other CNS-related symptoms (memory loss, headache), anti-spike, and nucleocapsid antibody titers, formerly used antiviral medications (e.g., remdesivir, favipiravir) (OR, 1.06; 95%CI, 1.004–1.118; *p* = 0.03). In this subgroup analysis, significantly higher PRDX3 level was detected in patients with post-COVID dizziness (*n* = 15) (median: 60.4 ng/ml, IQR: 49.4–68.3 vs 45.9, 35.5–57.6, *p* = 0.02). This specific association points to a nuanced role for PRDX3 in the post-COVID symptom complex, suggesting that while it may not correlate with the broader spectrum of long COVID symptoms, it could be particularly relevant to understanding and potentially managing specific symptoms like dizziness.

While initial findings highlighted PRDX3’s potential in reflecting oxidative stress within mitochondria, our results specifically connect elevated PRDX3 levels to the symptom of dizziness rather than a broader range of long COVID symptoms such as fatigue. This points to a more nuanced understanding of PRDX3’s role, necessitating a careful interpretation of its utility as a biomarker.

The association between increased PRDX3 levels and dizziness suggests a possible specific mitochondrial stress response in certain neurological contexts linked to long COVID. This finding underlines PRDX3’s sensitivity to particular pathological states—specifically, those involving enhanced oxidative stress, which is a known trigger for dizziness in various neurological disorders. The lack of a similar correlation between PRDX3 levels and other long COVID symptoms such as fatigue indicates that mitochondrial dysfunction, as measured by PRDX3, does not uniformly affect all symptoms of long COVID. This discrepancy highlights the need for a more symptom-specific approach in using PRDX3 as a mitochondrial biomarker.

Given the selective elevation of PRDX3 in patients experiencing dizziness, it is imperative to explore why this biomarker does not similarly elevate in relation to other symptoms. One hypothesis is that different long COVID symptoms may be driven by varying degrees of mitochondrial involvement or different pathophysiological processes within the mitochondria. For example, the mechanisms underlying fatigue might involve broader bioenergetic dysfunction not solely captured by oxidative stress markers such as PRDX3. This suggests the potential involvement of other mitochondrial functions or even non-mitochondrial pathways.

To further elucidate the role of mitochondrial dysfunction in long COVID, comprehensive studies should be conducted to expand biomarker panels, including a range of mitochondrial and oxidative stress markers to assess their correlation with a diverse spectrum of long COVID symptoms. Future studies should investigate the specific mitochondrial pathways that are implicated in different long COVID symptoms, which may help in understanding the selective impact on symptoms like dizziness. Further, clinical trials for mitochondria targeted therapies are needed to explore the effectiveness of antioxidants and mitochondrial support therapies in specifically treating the symptoms correlated with mitochondrial dysfunction, such as dizziness, to assess their therapeutic benefits and limitations.

## Potential therapeutic implications for mitochondrial health in long COVID

The recognition of the important role of mitochondrial dysfunction in long COVID opens new avenues for therapeutic intervention aimed at restoring mitochondrial health and function [19, 20]. Effective management of mitochondrial dysfunction and restoration of cellular energetics could potentially alleviate some of the persistent symptoms experienced by long COVID patients, such as fatigue, cognitive impairment, and muscle weakness. Strategies to improve mitochondrial function involve a combination of pharmacological interventions, lifestyle modifications, and nutritional support. Antioxidants, such as coenzyme Q10, MitoQ, N-acetylcysteine, resveratrol, and alpha-lipoic acid, have been suggested to reduce oxidative stress in mitochondria, thereby improving their function [148–151].

Additionally, compounds like L-carnitine, which facilitate fatty acid transport into mitochondria for energy production, could also prove beneficial [152, 153]. NAD^+^ boosters, such as nicotinamide riboside (NR) and nicotinamide mononucleotide (NMN), have garnered attention for their potential to enhance mitochondrial function by increasing the levels of nicotinamide adenine dinucleotide (NAD^+^), a critical coenzyme involved in cellular energy production and repair processes [132, 154–159]. By boosting NAD^+^ levels, these supplements may help counteract mitochondrial dysfunction and improve energy metabolism, offering a promising therapeutic avenue for conditions like long COVID [160]. Some of these compounds, including Q10 [67, 161, 162], MitoQ (NCT05373043), alpha-lipoic acid [163], nicotinamide riboside (NCT05703074), resveratrol (NCT05601180), are currently being tested in clinical trials to assess their effectiveness in treating patients with long COVID, reflecting the growing interest in targeting mitochondrial dysfunction as a therapeutic strategy for this condition. Exercise and physical rehabilitation programs tailored to long COVID patients may help enhance mitochondrial biogenesis, promoting the growth and division of existing mitochondria [164–168]. Nutritional interventions focusing on a diet rich in nutrients known to support mitochondrial health, including omega-3 fatty acids, B vitamins, and minerals like magnesium, could further support recovery.

Currently, treatments aimed at improving mitochondrial function are being explored in the context of various chronic diseases, including neurodegenerative disorders and metabolic syndromes. For instance, mitochondrial-targeted antioxidants have shown promise in reducing symptoms and improving quality of life in conditions characterized by mitochondrial dysfunction. While direct evidence of their efficacy in long COVID is still emerging, these treatments offer a plausible therapeutic approach given the shared underlying mechanisms of mitochondrial impairment.

Future research should focus on the development of targeted therapies that can directly enhance mitochondrial function or protect mitochondria from damage. This includes the exploration of novel pharmacological agents that can increase mitochondrial biogenesis, enhance their energy production capabilities, or reduce mitochondrial-related inflammation and apoptosis. Clinical trials specifically designed to evaluate the efficacy of mitochondrial-targeted therapies in long COVID populations are critically needed. Additionally, the identification of biomarkers indicative of mitochondrial dysfunction could help tailor therapeutic approaches to individual patient needs, improving outcomes.

Moreover, understanding the interaction between mitochondrial dysfunction and other pathophysiological processes in long COVID, such as immune dysregulation and endothelial dysfunction, could lead to the development of combination therapies that address multiple facets of the disease. Integrative approaches that combine pharmacological treatments with lifestyle and nutritional interventions may offer the most promise for comprehensive management of long COVID, aiming not only to alleviate symptoms but also to restore overall health and well-being.

## Challenges and future directions

Diagnosing and studying mitochondrial dysfunction in long COVID presents numerous challenges. The condition’s multifaceted symptomatology and the absence of standardized diagnostic criteria for mitochondrial dysfunction complicate the identification and treatment of affected individuals. Furthermore, the need for longitudinal studies is paramount to unravel the long-term implications of mitochondrial dysfunction in long COVID patients. Studies focusing on the biochemical pathways involved in mitochondrial energy production and oxidative stress in post-COVID-19 patients are warranted. These would include assessments of mitochondrial DNA, electron transport chain activity, and oxidative damage markers to better understand the extent and nature of mitochondrial dysfunction. Such studies are essential to understand the persistence of symptoms over time and their impact on patients’ quality of life. Research aimed at identifying and characterizing autoantibodies against mitochondrial antigens in long COVID patients could help establish a link between autoimmunity and persistent symptoms, guiding the development of immunomodulatory treatments. Identifying potential biomarkers for the early detection and monitoring of mitochondrial dysfunction in long COVID is another critical area of research. Biomarkers could offer invaluable insights into the disease’s progression and response to treatment, facilitating personalized therapeutic approaches.

## Conclusion

The exploration of mitochondrial dysfunction in the context of long COVID has unveiled a critical aspect of the condition’s underlying pathology. This review underscores the pivotal role that mitochondrial impairment plays in contributing to the wide array of symptoms associated with long COVID, ranging from chronic fatigue and cognitive disturbances to metabolic and vascular dysfunctions. Mitochondrial health emerges as a promising target for alleviating some persistent symptoms experienced by long COVID patients. The potential for interventions—ranging from pharmacological treatments to lifestyle modifications, exercise, and dietary approaches—offers hope for those struggling with this condition. Yet, our journey towards fully understanding and effectively addressing long COVID is far from over. Significant gaps remain in our comprehension of how SARS-CoV-2 infection precipitates mitochondrial dysfunction and the manner in which this dysfunction perpetuates the wide range of long COVID symptoms. Addressing these challenges and moving forward requires a multidisciplinary collaboration to delve deeper into the role of mitochondrial dysfunction in long COVID. Such endeavors will not only broaden our understanding but also expedite the development of targeted and effective treatments, ultimately offering hope and improved outcomes for patients worldwide.

## Data Availability

The datasets used and or analyzed in the current study are available from the corresponding author upon reasonable request.
